# Generation of spatiotemporally tailored terahertz wavepackets by nonlinear metasurfaces

**DOI:** 10.1038/s41467-019-09811-9

**Published:** 2019-04-16

**Authors:** Shay Keren-Zur, Mai Tal, Sharly Fleischer, Daniel M. Mittleman, Tal Ellenbogen

**Affiliations:** 1 Department of Physical Electronics, School of Electrical Engineering, Tel-Aviv University, 6997801 Tel Aviv, Israel; 2 Center for Light-Matter Interaction, Tel-Aviv University, 6779801 Tel-Aviv, Israel; 3 Raymond and Beverly Sackler Faculty of Exact Sciences, School of Physics & Astronomy, Tel-Aviv University, 6779801 Tel-Aviv, Israel; 4 Raymond and Beverly Sackler Faculty of Exact Sciences, School of Chemistry, Tel Aviv University, 6997801 Tel Aviv, Israel; 5 School of Engineering, Brown University, Providence, RI 02912 USA

## Abstract

The past two decades have witnessed an ever-growing number of emerging applications that utilize terahertz (THz) waves, ranging from advanced biomedical imaging, through novel security applications, fast wireless communications, and new abilities to study and control matter in all of its phases. The development and deployment of these emerging technologies is however held back, due to a substantial lack of simple methods for efficient generation, detection and manipulation of THz waves. Recently it was shown that uniform nonlinear metasurfaces can efficiently generate broadband single-cycle THz pulses. Here we show that judicious engineering of the single-emitters that comprise the metasurface, enables to obtain unprecedented control of the spatiotemporal properties of the emitted THz wavepackets. We specifically demonstrate generation of propagating spatiotemporal quadrupole and few-cycles THz pulses with engineered angular dispersion. Our results place nonlinear metasurfaces as a new promising tool for generating application-tailored THz fields with controlled spatial and temporal characteristics.

## Abstract (web summary)

While the terahertz range has become increasingly important for a wide range of applications, efficient sources with bespoke output characteristics are still lacking. Here, Keren-Zur *et al*. show that engineering of a metasurface’s individual elements allows control of the spatiotemporal properties of the emitted terahertz radiation.

## Introduction

The terahertz spectral band (10^11^–10^13^ Hz) is located between the infrared and the microwave frequency regimes. The generation and detection of THz waves is highly desirable as it allows probing and even manipulating low-energy degrees of freedom such as rotations in molecules^1^, collective vibrations in molecular crystals^2^, hydrogen bond frameworks^3^, excitons in semiconductors, spin-waves in magnetic materials^4^, to give just a few examples. Since a wide variety of materials are transparent to THz radiation, it also provides an excellent tool for high-resolution imaging and spectroscopy through optically opaque samples^5^. Moreover, single-cycle THz pulses further permit time-resolved imaging and depth-resolved tomography^6^ for important biomedical and security applications^7^ and even for studies of art^8^. Furthermore, the THz band can also support fast wireless communication links beyond fifth-generation protocols^9^.

While vast advancements over the past two decades yielded a variety of methods for generation and detection of THz waves^10–12^, their spatial and temporal manipulation remains highly challenging. In fact, optical elements such as wave-plates, lenses, and beam-shaping devices that are commercially available in the entire optical range are hardly available in the THz band. In addition, simple passive elements that work well for continuous (narrow band) THz waves impose severe compromise when using single- or few-cycle pulses due to dispersion and multiple reflections that are very common in THz measurements. In particular, retardation elements cannot be used for single-cycle beam shaping, since they cause spatial time delay to the pulse. Nevertheless, the spatial and temporal structuring of THz waves may be highly beneficial for many emerging THz applications, including mode multiplexing in THz communications^13^, super-resolution and complex imaging techniques in the THz domain^14^, and for spatially resolved THz spectroscopy. Therefore, it is vital to develop new methods to manipulate THz waves, which will push forward emerging THz technologies. In this work, we present a promising method to generate single- and few-cycle THz wavepackets with designed spatiotemporal structures by using spatially engineered nonlinear plasmonic metasurfaces.

Engineered metasurfaces and metamaterials^15^ were demonstrated to be useful for development of various new types of optical THz elements. Engineered absorption^16^, polarization conversion^17^, active phase modulation^18^, and even nonlinear effects^19,20^ were observed in THz metamaterials. In addition, optical metasurfaces were proven to enhance the THz emission from photoconductive antennas^21^. However, full spatial and temporal control was not demonstrated yet. Few recent works studied the possibility to generate THz radiation from optically excited plasmonic metasurfaces. Initially, broadband THz generation by ponderomotive acceleration of photoelectrons was reported^22,23^, and more recently broadband THz generation by optical rectification (OR) on nonlinear metasurfaces (NLMSs) was also demonstrated and studied^24–26^. In the latter mechanism, the generation efficiency that was demonstrated from ultrathin metasurface was comparable and even exceeded that of OR in conventional sub-mm thick THz crystals, such as ZnTe and GaP. Therefore, in this work we utilize the demonstrated large structural OR effect as a source for the THz generation.

## Results

### Terahertz emission from NLMSs

**Fig. 1:**
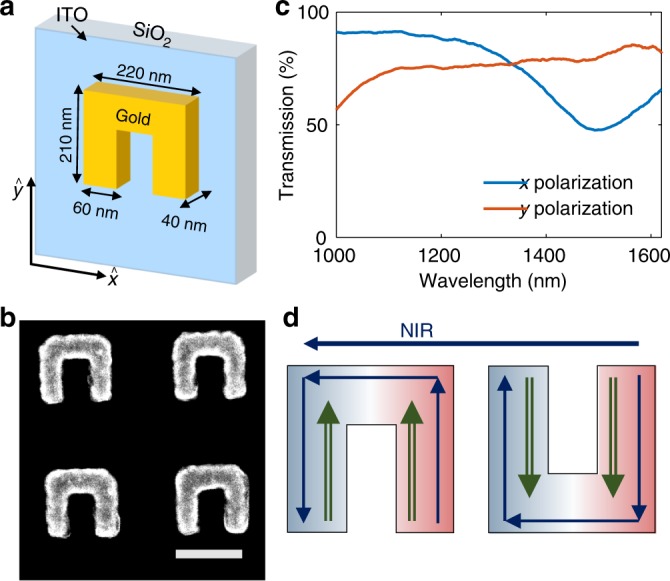
Metasurface characteristics. **a** Structure of the split-ring resonators (SRRs) that were used as basic inclusion for the nonlinear metasurface (NLMS). **b** Scanning electron microscope image of the fabricated SRRs. Scale bar is 200 nm. **c** Transmission plot for horizontal and vertical polarizations (relative to the SRR orientation in **a**), showing a dip in the transmission around 1500 nm in the horizontal polarization, associated with the magnetic resonance of the SRR. **d** Illustration of excited linear and nonlinear currents in SRR. Excitation with near infrared (NIR) pulse (blue) polarized along the *x* axis excites surface currents along the SRR. As a result, nonlinear currents (green) are generated along the arms of the SRR, in the *y* axis direction

The metasurfaces that we use are constructed of gold split-ring resonators (SRRs) fabricated on indium-tin-oxide (ITO)-coated glass (Fig. 1a, b and “Methods”). These building blocks act as deep subwavelength THz dipoles, ~1000 times smaller than the emitted wavelength. The ability to design each of them independently on the metasurface allows unparalleled control over the THz wavepacket emitted from the metasurface. The linear transmission through the metasurface (Fig. 1c) shows a dip at 1500 nm for polarization parallel to the base of the SRR, due to localized surface plasmon resonance of the SRR^27^. It was shown that this excitation configuration gives rise to nonlinear surface currents oscillating along the arms of the SRR^24,28^. These currents act as the nonlinear source for the emitted electromagnetic field, as described in Fig. 1d. In addition, for the case of second-harmonic generation (SHG), it was demonstrated that inversion of the SRR relative to its base leads to local nonlinear emission with an opposite phase^29^. This concept, along with its expansion to nonlinear phase gradient and geometrical phase^30–32^, allows versatile control over the SHG process for achieving a variety of intriguing functionalities, including nonlinear focusing, nonlinear beam shaping, and nonlinear holographic multiplexing of the SHG^29,30,33–36^.

**Fig. 2:**
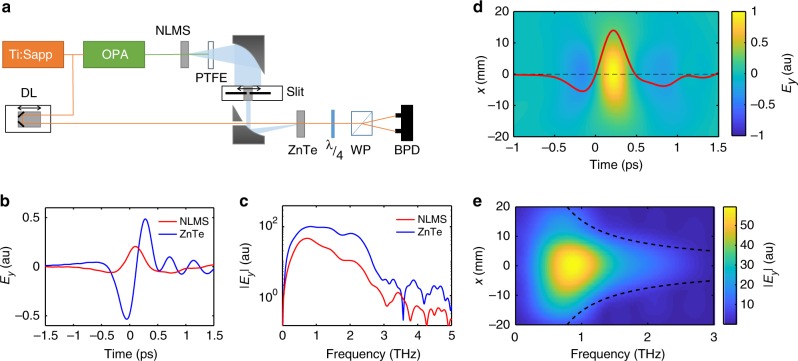
Terahertz emission from uniform nonlinear metasurfaces (NLMS). **a** Experimental set-up. Ti:Sapp amplified titanium sapphire laser, OPA optical parametric amplifier, PTFE teflon filter, DL delay line. ZnTe 0.5 mm ZnTe (110-cut) crystal, *λ*/4 quarter wave plate, WP Wollaston prism, BPD balanced photodiode. **b** Time domain spectroscopy (TDS) signal of terahertz (THz) pulse emitted from an NLMS (red line) and from a 0.1 mm ZnTe (110-cut) crystal under the same excitation conditions (blue line). The ripples following the single-cycle pulse emitted from the ZnTe are due to phonon absorption in the ZnTe crystal. **c** Emission spectrum from a uniform NLMS and ZnTe. **d** Spatiotemporal measurement of THz pulse emitted from NLMS, plotted in the time domain. The temporal cross-section at the center of the beam is shown in red. **e** Frequency domain representation of the measurement in **d**. The dashed lines mark the expected frequency-dependent diffraction profile of a beam emitted from a 1 mm wide emitter

We excite the metasurface with near-infrared (NIR) femtosecond pulses (“Methods”), which leads to single-cycle THz field generation^24^. The emitted signal is characterized by a time-domain-spectroscopy system (TDS) based on electro-optic sampling^10,37^, (Fig. 2a and “Methods”). In order to spatially characterize the THz beam, we raster scan the collimated beam in one dimension with a 7-mm wide slit that is mounted onto a linear translation stage.

Figure 2b shows the time-resolved THz emission from a uniform 1 × 1 mm^2^ metasurface, yielding a single-cycle THz pulse with <1 ps duration and peak field amplitude comparable to that generated from a 0.1-mm ZnTe crystal, as also reported previously^24^. This allows comparison with other THz generation methods^38^. Figure 2c shows the frequency domain of Fig. 2b. Figure 2d shows the measured spatiotemporal profile of the emitted THz beam. The measured beam exhibits a constant phase along the transverse direction as expected from an emitter with a spatially uniform nonlinear response. Figure 2e shows the Fourier transform of the measurement in Fig. 2d from time to frequency domain, representing the spectral distribution of the emitted THz beam. The peak intensity of the pulse is around 0.85 THz and the spectral diffraction is inversely proportional to the frequency, corresponding to the diffraction from a 1-mm wide emitter. See Supplementary Notes 1 (and “Methods” section) for the calculated spatiotemporal and spatiospectral emission patterns according to broadband beam-propagation technique, which are in good agreement with experimental data.

### Generation of single-cycle Hermite–Gauss wavepacket

**Fig. 3:**
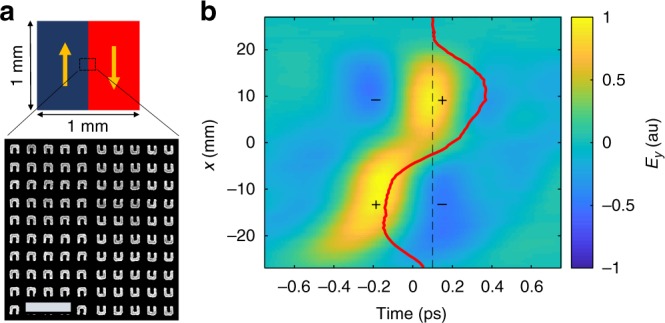
Generation of single-cycle Hermite–Gauss wavepacket. **a** Structure of nonlinear metasurface (NLMS) for generation of Hermite-Gauss(1,0) (*HG*_10_) single-cycle terahertz beam. Arrows indicate the orientation of the split-ring resonators. Scanning electron microscope image of the center of the NLMS is shown in the enlarged picture. Scale bar is 1 μm. **b** Spatiotemporal profile of the single cycle *HG*_10_ beam emitted from the NLMS in **a**. Spatial field profile along the dashed line is plotted in red. The ± signs mark the lobes of spatiotemporal quadrupole structure formed on the *xz* plane

Next, we harness the capability to locally engineer the nonlinear response at the single THz dipole-emitter level to demonstrate spatiotemporal shaping of the THz emission. As a simple example, we design an NLMS that generates single-cycle Hermite–Gauss(1,0) (*HG*_10_) THz wavepacket. To achieve this goal, we fabricate a 1 × 1 mm^2^ metasurface in which one half has an opposite orientation of the SRRs relative to the other half, as presented in Fig. 3a. Consequently, the phase of the THz dipoles is manipulated in space^29,33^. When such metasurface is excited with a Gaussian NIR beam, the generated THz field distribution follows:

1 $$E_y^{{\mathrm{THz}}}\left( {x,y} \right) = {\cal{A}}_{yxx}{\mathrm{sign}}\left( x \right)\left| {E_x^{{\mathrm{NIR}}}} \right|^2e^{ - \frac{{x^2 + y^2}}{{w^2}}}$$

where $E_x^{{\mathrm{NIR}}}$ is the amplitude of the NIR incident beam polarized along the *x* axis, *w* is the beam waist, and *A*_*yxx*_ is the effective nonlinear-response-tensor element^39^ of a uniform metasurface constructed of SRRs with their base oriented along the *x* axis. Equation 1 can be expanded into two-dimensional Hermite–Gauss functions, with the highest contribution of the *HG*_10_ component, which propagates to the far-field. The measured spatiotemporal electric-field profile is shown in Fig. 3b. It can be seen that the phase of the measured wavepacket is inverted along the transverse *x* direction, as expected from an *HG*_10_ beam. It is important to note that using conventional THz phase plates for conversion of single-cycle *HG*_00_ pulses to higher order forms cannot be achieved. Even if highly broadband phase plates are engineered, they would impose a time delay to parts of the single-cycle pulse that will manifest in spatiotemporally distorted waves, instead of maintaining a temporally aligned pulse front.

Moreover and very interestingly, it can be seen that the single-cycle pulse with a transverse profile of *HG*_10_ forms a spatiotemporally confined quadrupole structure, which is moving along the propagation direction at the speed of light. Recently, there has been a lot of interest in generation of related nontrivial propagating modes of Maxwell’s equation, which also have a field component along the propagation axis when focused (Supplementary Note 2). These so-called “flying” wavepackets were predicted to be useful for particle acceleration and excitation of nontrivial interactions with interfaces and nanostructures^40,41^; however, to the best of our knowledge, these were not demonstrated experimentally until now. As shown in Supplementary Note 2, the demonstrated wavepacket corresponds very well to a polarized projection of a “flying doughnut”^40,42^.

### THz generation by nonlinear metamaterial photonic crystal (NLMPC)

The ability to manipulate the phase of the generated THz field also allows to construct THz NLMPCs^29^. It was shown that NLMPCs provide enhanced control over the generated light in various ways, including angular control of different frequency components and their propagation to different locations in space. For a one-dimensional periodic NLMPC with a lattice constant of *Λ*, each generated THz wavelength, *λ*_THz_, is directed according to the Raman–Nath diffraction (Supplementary Note 3):

2 $${\mathrm{sin}}\theta _m\left( {\lambda _{{\mathrm{THz}}}} \right) = \frac{{m\lambda _{{\mathrm{THz}}}}}{{\mathrm{\Lambda }}} + {\mathrm{sin}}\theta _{{\mathrm{in}}}$$

where *θ*_*m*_ is the wavelength dependent emission angle of the *m*th order and *θ*_in_ is the incidence angle of the exciting beam relative to the modulation axis (as depicted in Supplementary Fig. 3 and Supplementary Note 3). This frequency-dependent deflection of the emitted wave is highly attractive for the development of new single-shot frequency-domain THz spectrometers, instead of the conventional time domain spectroscopy, that requires a complex optical system. In this method, each frequency is deflected at a different angle. Therefore spatially resolved intensity measurement of the beam’s profile after transmission through the sample, corresponds with the spectral transmission. This is much like conventional spectrometers that operate in the ultraviolet–visible–IR regimes, only in this case at THz frequencies and without the use of diffraction gratings.

**Fig. 4:**
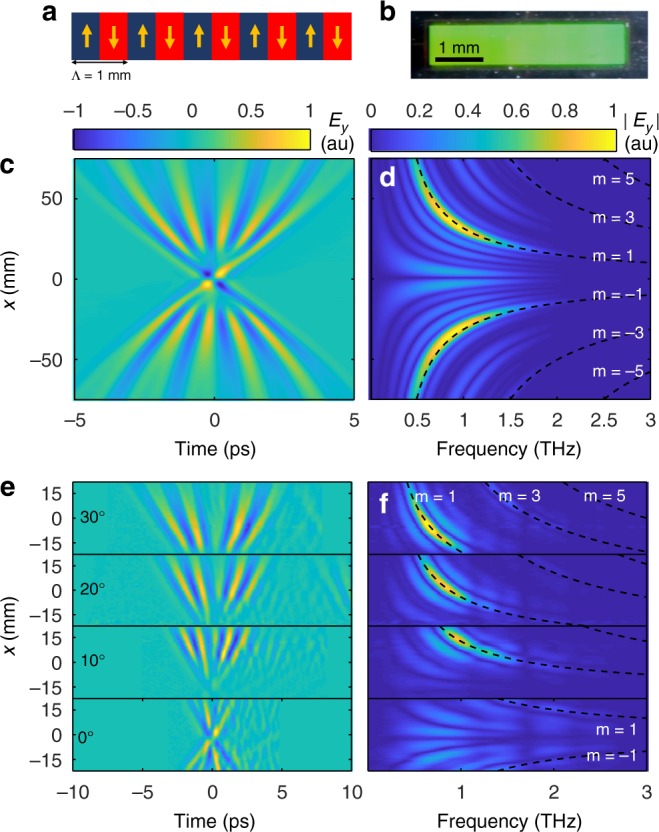
Terahertz generation from nonlinear metamaterial photonic crystals (NLMPC). **a** Structure of the fabricated NLMPC. Arrows indicate the orientation of the split-ring resonators, similarly to Fig. 3a. **b** Reflection image of the fabricated NLMPC. **c** Simulation of the spatiotemporal profile of the THz emission pattern from the NLMPC in **a** when excited by the near-infrared pulse at normal incidence. **d** Frequency domain spatial profile of **c**. Dashed lines mark the Raman–Nath diffraction orders. **e** Measured THz emission pattern from NLMPC fabricated according to **a**, with varying incidence angles, in the time domain and **f** in the frequency domain. Dashed lines indicate calculated Raman–Nath relation for different diffraction orders

To study the emission from THz NLMPC, we fabricated a sample of 5 × 1 mm^2^ with a 1-mm modulation period, as shown in Fig. 4a, b. Figure 4c presents the simulated time domain profile of the THz field emitted from a NLMPC of these measures. It can be seen that the field at each transverse location of the collimated beam (i.e., corresponding to emission angle) forms a pulse with varying carrier frequency. The diffracted pulse is broadened in time, due to the angular dispersion given by the periodic structure^43^. As a result, the single-cycle pulse is transformed into a few-cycle pulse, a desirable capability in its own right^44^. Note that the number of cycles in each diffracted pulse is equal to the number of periods in the NLMPC (Supplementary Note 4).

The simulated frequency domain profile in Fig. 4d shows that the THz wavelength-dependent diffraction follows the Raman–Nath relation with a sinc-like bandwidth broadening due to the finite size of the NLMPC, which is also convoluted with the NLMS emission spectrum. The experiment is performed by illuminating the NLMPC with a 7-mm full-width at half maximum Gaussian beam. To overcome the limited NA of our system (NA = 0.25) and capture the full diffraction pattern, we excite the sample with varying incidence angles, *θ*_in_, and measure the corresponding collected THz profile. The measurements presented in the time domain and the frequency domain for the different incidence angles are shown in Fig. 4e, f, respectively. The measured signals are in a good agreement with the simulated field distribution calculated in Fig. 4c, d, showing the desired spectral diffraction pattern according to the calculated Raman–Nath diffraction orders 1, 3, 5, −1 (dashed lines in Fig. 4f). The calculated emission patterns for varying incident angle are presented in Supplementary Note 1 and are in good agreement with the experimental results of Fig. 4.

The unique capability to design the nonlinear response at each position on the NLMS provides additional advantageous functionalities. For example, the amplitude of the generated THz emission from each point on the NLMS can be continuously controlled by transformation of the SRR geometry^33,45^ or by changing the local concentration of the emitters. This enables us to tailor the carrier-envelope of the few-cycles pulse and obtain flexible THz pulse shaping capabilities (see Supplementary Note 5). In addition, according to the generation scheme shown in Fig. 1d, rotation of the SRR effectively changes the THz dipole polarization, thus changing the local emission polarization. However, one must take into account the excitation scheme and the full tensorial behavior of the nonlinear response.

## Discussion

In conclusion, we demonstrate a novel method for generation of structured single-cycle THz wavepackets from engineered NLMS. Such NLMS can replace conventional nonlinear crystals in THz generation systems to provide new functionalities without sacrificing efficiency, avoid phononic interactions or phase mismatch considerations, and potentially obtain larger THz bandwidths^25,26^. This unprecedented capability, which overcomes several limitations of conventional THz generation and manipulation schemes, opens the door for advanced THz technologies, such as beam multiplexing, structured-light THz imaging, single-shot spectroscopy, THz holography, particle manipulation, and structured THz light–matter interactions. It also allows the investigation of the intriguing confined spatiotemporally structured fields such as the so-called flying electromagnetic doughnuts^40,41^, in addition to THz Bessel beams and pulses carrying angular orbital momentum. Lastly, in addition to interesting new applications, the field of THz generation and shaping by NLMS holds many new fundamental opportunities yet to be studied thanks to the versatile control over the local and collective nonlinear interactions on metasurfaces. Further investigation of the full nonlinear response tensor of the NLMS will add to the toolbox of THz wavepacket shaping the ability to obtain control over the spatial polarization, amplitude, and continuous phase. The physical mechanism of the nonlinear interaction itself can be additionally studied to shed new light on ways to significantly enhance their THz generation efficiency, e.g., based on designing the single element geometry^28,45^, coupling to hetero-structures^25,46,47^, and by the study of collective effects on the NLMS^48,49^.

## Methods

### NLMS fabrication

An indium tin oxide-coated (~20-nm thick) glass substrate was cleaned by sonication in acetone and isopropyl alcohol (IPA). The clean substrate was spin-coated with polymethyl methacrylate(PMMA) resist and baked at 180 °C on a hotplate for 1 min. The SRR arrays were patterned by an electron beam lithography system (Raith 150 II) using modulated beam–moving stage technique at 20 kV and 20 μm aperture. The patterned sample was developed in cooled (4 °C) MIBK/IPA 1:3 for 1 min and dried under N_2_ stream. Three-nm Ti was evaporated as an adhesion layer, followed by 37 nm Au. The photoresist was lifted-off in acetone.

### Generation and measurement of THz beams

Amplified titanium–sapphire laser (Spectra-physics Solstice ACE), generating pulses of 35 fs with 800 nm central wavelength, 2 kHz repetition rate, and 3.5 mJ per pulse, was used as the laser source. The pulse was split by a beam sampler (~1% reflection) to generate a probe beam and the rest converted by an optical parametric amplifier (TOPAS) to around 1550 nm for the pump beam. Half and quarter broadband wave plates, Glan laser polarizer, and wire grid polarizer were used to attenuate the pump beam and control its polarization state. A mechanical chopper, synchronized to the amplified laser system sub-harmonic, chopped the pump beam at 1 kHz. The NLMS samples were illuminated by the pump beam with a typical average power (after chopping) of 15 mW and ~1.7 mm beam diameter. The samples were placed such that the NLMS were facing the detector. After passing through the NLMS, pump beam was filtered by a 5-mm-thick Teflon slab placed after the sample, and the THz emitted from the sample was collimated by an off-axis parabolic mirror with 4” focal length and 2” diameter. The collimated beam was polarized by a THz wire grid polarizer and passed through a 7-mm slit, which was sufficient for getting high spatial resolution and yet large enough to avoid significant diffraction effects. The slit was mounted over a translation stage moving along the *x* axis and the orientation of the slit was along the *y* axis. The stage was used in order to raster scan the beam profile. The THz wave was then focused by an off-axis parabolic mirror with a hole for the probe beam onto a 0.5-mm-thick ZnTe crystal used as an electro-optic crystal^37^. A delay line was used for temporal scan of the probe pulse along the THz pulse duration as they coincide at the electro-optic crystal. Owing to the slow change of the THz wave relative to the probe pulse duration, the THz field acts effectively as a DC field in the electro-optic crystal and induces birefringence that results in the rotation of the probe polarization. The probe beam passes through a quarter wave plate and spatially separated into two cross-polarized beams by a Wollaston prism followed by a balanced photodiode for detection. The balanced signal is amplified by a lock-in amplifier (Stanford research systems SR830) locked to the chopper frequency. Labview was used to control the delay line, motorized stage, mechanical chopper, and lock-in amplifier in order to perform the measurements.

### THz propagation simulations

The simulations were based on beam propagation technique and were calculated using MATLAB. The broadband pulse was defined by a spectrum matching the measured THz signal. The electric field over the excited NLMS plane was defined for each frequency to be its spectral amplitude with a sign matching the orientation of the SRR on the NLMS and zero outside the NLMS. The spatial Fourier components of the field *E*(*k*_*x*_) were propagated along the propagation direction, *z*, for each temporal frequency, *f*, by phase addition of *k*_*z*_*z* with $k_z = \sqrt {\left( {\frac{{2\pi f}}{c}} \right)^2 \,-\, k_x^2}$. The collimation of the beam by a parabolic mirror was simulated by phase addition. The propagated spatiospectral structure was then used to reconstruct the spatiotemporal field by inverse Fourier transform in time and space domains.

## Supplementary information

Supplementary Information

## Data Availability

All relevant data are available from the authors.

## Code availability

All relevant codes are available from the authors.
