# Supplementary material for: Generation of spatiotemporally tailored terahertz wavepackets by nonlinear metasurfaces
Source: Nat Commun. 2019 Apr 16;10:1778. doi: 10.1038/s41467-019-09811-9 (PMC6467996; doi:10.1038/s41467-019-09811-9)
Supplement: Supplementary file 1 — Supplementary Information [file 41467_2019_9811_MOESM1_ESM.pdf]

Supplementary Information

Generation of spatiotemporally tailored THz wavepackets by nonlinear metasurfaces

Shay Keren-Zur, Mai Tal, Sharly Fleischer, Daniel M. Mittleman, Tal Ellenbogen

Supplementary Note 1

Comparison of experimental results with beam propagation simulations

Broadband beam propagation calculations (see Methods section in the main text) were used to verify the THz emission pattern from the NLMS. Supplementary figures 1a-b show the expected emission pattern in time and frequency domain respectively from a 1mm wide uniform NLMS, in comparison to the measurements shown in Fig 2d-e of the main text. Supplementary Figures 1c-d show the expected emission pattern from the NLMPC as presented in Fig. 4c-d of the main text, including the effect of the varying angle of incidence, as was measured and presented in Fig. 4e-f of the main text.

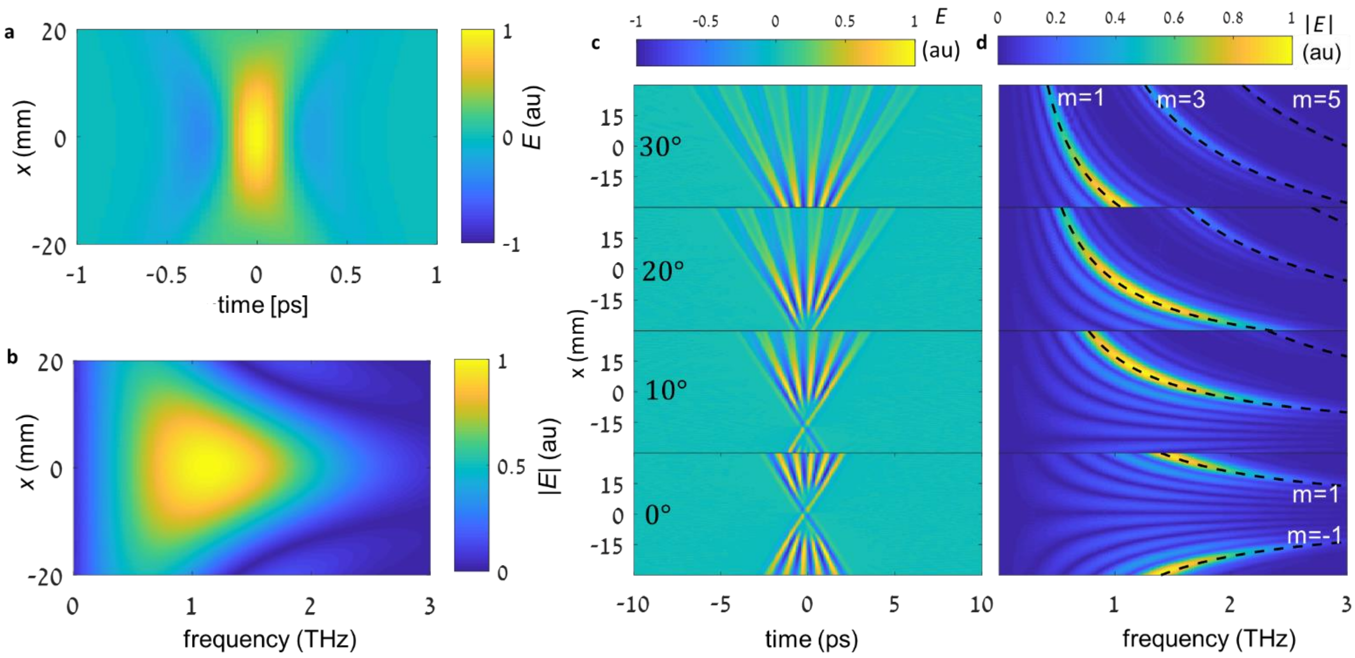

Supplementary Figure 1 Simulated terahertz emission patterns. **a** Spatiotemporal emission pattern from a 1mm uniform NLMS, as calculated by broadband beam propagation simulation. **b** Simulated spatiospectral emission from $1mm$ uniform NLMS. **a** and **b** are presented in comparison to the measured wavepacket shown in Fig. 2d-e of the main text. **c** Simulated spatiotemporal emission pattern from NLMPC as shown in Fig. 4 of the main text, with varying incident angles $(0°, 10°, 20°, 30°)$ in comparison to the actual measurements. **d** Spatiospectral emission pattern, complementary to **c**.

Supplementary Note 2

Flying doughnuts

Maxwell's equations have many well-known solutions that have separable temporal and spatial structures. In addition, there is a group of solutions that draw a lot of attention, in which the spatial and temporal components are inseparable[1–4]. One of the most known wavepackets among this group are the so-called "Flying doughnuts"(FD)[2,5,6]. These are single-cycle pulses with a

doughnut-like electromagnetic profile, which move at the speed of light. FD are also known for having a field component along the propagation direction when focused, which can be used for electron acceleration and intriguing light-matter interaction in the nanometric scale. The electromagnetic field of FD was derived before[1,2] and was shown to be:

$$E_\theta = -4if_0\sqrt{\frac{\mu_0}{\epsilon_0}}\rho\frac{q_1+q_2-2ict}{\left[\rho^2+\left(q_1+i(z-ct)\right)\left(q_2-i(z+ct)\right)\right]^3} \tag{1}$$

$$H_\rho = 4if_0\rho\frac{-q_1+q_2-2iz}{\left[\rho^2+\left(q_1+i(z-ct)\right)\left(q_2-i(z+ct)\right)\right]^3} \tag{2}$$

$$H_z = -4f_0\frac{\rho^2-\left(q_1+i(z-ct)\right)\left(q_2-i(z+ct)\right)}{\left[\rho^2+\left(q_1+i(z-ct)\right)\left(q_2-i(z+ct)\right)\right]^3} \tag{3}$$

In this work we present a single-cycle HG_{10} "flying" field, which also carries a longitudinal polarization components when focused. Moreover, it has the form that is similar to the *y*-polarization component of an FD. Supplementary Figure 2 shows the resemblance between the profile of the HG_{10} wavepacket profile as simulated with accordance to the NLMS presented in Fig. 3, and the cross-section of the E_y component of a FD along the *x*-axis at $y = 0$. A full form FD can be generated by superposition of HG_{10} wavepacket with its 90 degrees rotated counterpart.

Another way to achieve this will be by a more complex design of the NLMS, which will include manipulation of the locally emitted polarization state, e.g., by circularly symmetric rotation of the SRRs along the metasurface.

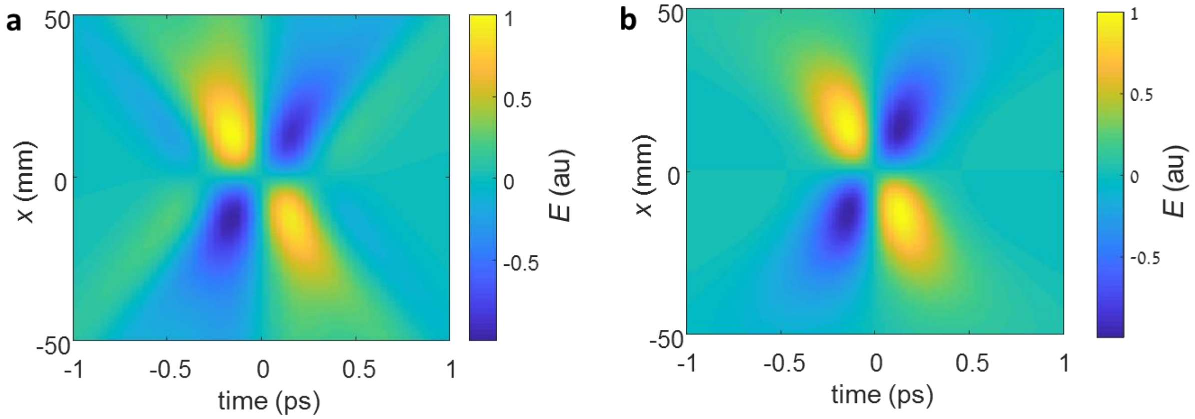

Supplementary Figure 2 Hermite-Gauss (1,0) wavepacket compared with theoretical "flying doughnut". **a** Simulated spatiotemporal profile of HG_{10} THz wavepacket corresponding with HG_{10} THz generation with NLMS as described in Figure 3 of the main text. **b** E_y component spatio-temporal profile of the x- cross section at $y = 0$ of a "flying doughnut" according to Supplementary Equation 1 with $z = 0$, $q_1 = 8 \times 10^{-5}$, $q_2 = 11$.

Supplementary Note 3

Nonlinear Raman-Nath diffraction in THz generation

For a simple, one-dimensional nonlinear periodic structure along the x-axis with a lattice constant of Λ, the energy conservation condition in optical rectification process is:

$$|\mathbf{k}_1 - \mathbf{k}_2| = \left|\mathbf{k}^{(m)}{}_{\mathrm{THz}}\right| \tag{4}$$

where $\mathbf{k}_1$ and $\mathbf{k}_2$ are the the wave-vectors of the broadband NIR beam. $\mathbf{k}_{\mathrm{THz}}$ is the emitted THz wave-vector and m is an odd number marking the diffraction order, as also depicted in Supplementary Figure 3. For NLMPC with 0.5 duty cycle only odd Fourier components construct the spatial structure of the nonlinear response tensor, therefore the emitted THz wave is directed only to the odd diffraction orders.

The momentum conservation condition along the x-axis requires:

$$k_{1x} - k_{2x} + \frac{2\pi m}{\Lambda} = k_{\mathrm{THz,x}}^{(m)} \tag{5}$$

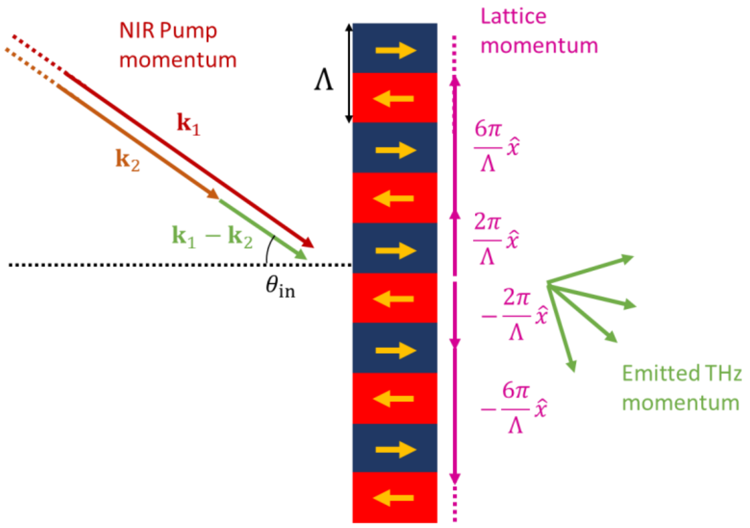

Supplementary Figure 3 Momentum matching scheme for THz generation in NLMPC. $\mathbf{k}_{1,2}$ are the momenta of the incidence NIR frequency components that pump the nonlinear metasurface, with incidence angle of θ_{in}. $\mathbf{k}_1 - \mathbf{k}_2$ is the THz wave momentum generated by the OR process. Combined with the lattice momenta given by the periodic structure of the NLMPC, the emitted THz wavepacket is deflected.

The emission THz angle is:

$$\sin\theta_m = \frac{k_{\mathrm{THz,x}}^{(m)}}{\left|\mathbf{k}_{\mathrm{THz}}^{(m)}\right|} \tag{6}$$

Which gives, according to Supplementary Equations (4)-(6) the Raman-Nath diffraction for THz wavelength (λ_{THz}) emitted by an NLMPC:

$$\sin\theta_m\,(\lambda_{\mathrm{THz}}) = \frac{m\lambda_{\mathrm{THz}}}{\Lambda} + \sin\theta_{\mathrm{in}} \tag{7}$$

where θ_m is the diffraction angle of the m^{th}order and θ_{in} is the NIR incidence angle relative to the modulation direction.

Supplementary Note 4

Calculation of number of cycles in a diffracted pulse

The profile of the THz electric field on the NLMPC plane can be described by:

$$E_{\mathrm{THz}}(x) = E_0 \Pi\left(\frac{x}{L}\right) \mathcal{S}\left(\frac{x}{\Lambda}\right) \tag{8}$$

where $\Pi(x)$ is the unit-box function, given by the finite size of the NLMPC, L is the NLMPC length, $\mathcal{S}$ is a square wave function and Λ is the period of the NLMPC.

The spatial frequency distribution is given by its Fourier transform:

$$E(k_x) = \mathcal{FT}\{E_{\mathrm{THz}}(x)\} \tag{9}$$

$$= \frac{2LE_0}{i\pi} \sum_{m=\pm1,\pm3,\pm5..} \frac{1}{m} (\mathrm{sinc}\left(\left(k_x - \frac{2\pi m}{\Lambda}\right)\frac{L}{2}\right) \mathrm{sign}(m)$$

Each m term denotes a diffraction order. While an infinite grating is described by $\delta\left(k_x - \frac{2\pi m}{\Lambda}\right)$, here the diffraction order is convoluted with a $sinc$ function with a central spatial frequency $k_{x,\mathrm{c}} = \frac{2\pi m}{\Lambda}$ width of $\Delta k_x \approx \frac{2}{L}$, due to the finite size of the NLMPC.

The spatial bandwidth of the diffraction pattern of a certain temporal frequency, can be used to calculate the temporal bandwidth at a certain diffraction angle:

$$\Delta f|_{f_\mathrm{c}} = \left.\frac{\partial f}{\partial \theta}\right|_{f_\mathrm{c}} \left.\frac{\partial \theta}{\partial k_x}\right|_{f_\mathrm{c}} \Delta k_x \tag{10}$$

With θ as the Raman-Nath diffraction angle:

$$\theta = \mathrm{asin}\left(\frac{k_x}{k}\right) \approx \frac{k_x}{k} = \frac{k_x c}{2\pi f} \tag{11}$$

With k as the emitted wave vector length - $k = \frac{2\pi f}{c}$, and consequently $f = \frac{c k_x}{2\pi\theta}$.

The bandwidth of the pulse at a specific emission angle is therefore:

$$\Delta f|_{f_{\mathrm{c}}} = \left(-\frac{ck_x}{2\pi\theta^2}\right)\left(\frac{c}{2\pi f_c}\right)\frac{2}{L} = \frac{f_{\mathrm{c}}}{k_x}\frac{2}{L} = \frac{f_{\mathrm{c}}}{\pi}\frac{\Lambda}{L} \tag{12}$$

In addition, the number of cycles in a diffracted pulse (N_{c}) is proportionate to:

$$N_{\mathrm{c}} \propto \frac{f_{\mathrm{c}}}{\Delta f} \propto \frac{L}{\Lambda} \tag{13}$$

Therefore N_{c} is propotional to the number of periods,$N_{\mathrm{p}} = \frac{L}{\Lambda}$. For the case described in the main text, it is shown that for $\Lambda = L$, i.e. $N_{\mathrm{p}} = 1$ the NLMS emits a single cycle- $N_{\mathrm{c}} = 1$. Consequently we get that $N_{\mathrm{c}} = N_{\mathrm{p}}$.

Supplementary Figures 4a-c present simulations of spatiotemporal profile of THz pulse emitted from a NLMPC with a period of $\Lambda = 1$mm and $N_{\mathrm{p}} = \frac{L}{\Lambda} = 3{,}5{,}10$. It can be seen clearly that the number of the cycles in the emitted pulse (N_{c}) is equal to N_{p}. Supplementary Figures 4d-f shows the spatio-spectral profile corresponding to Supplementary Figures 4a-c, presenting a diffraction bandwidth that narrows with increased size of the metasurface L. This is due to the opposite relation between NLMPC size with the sinc function width which is convoluted with the Raman-Nath relation.

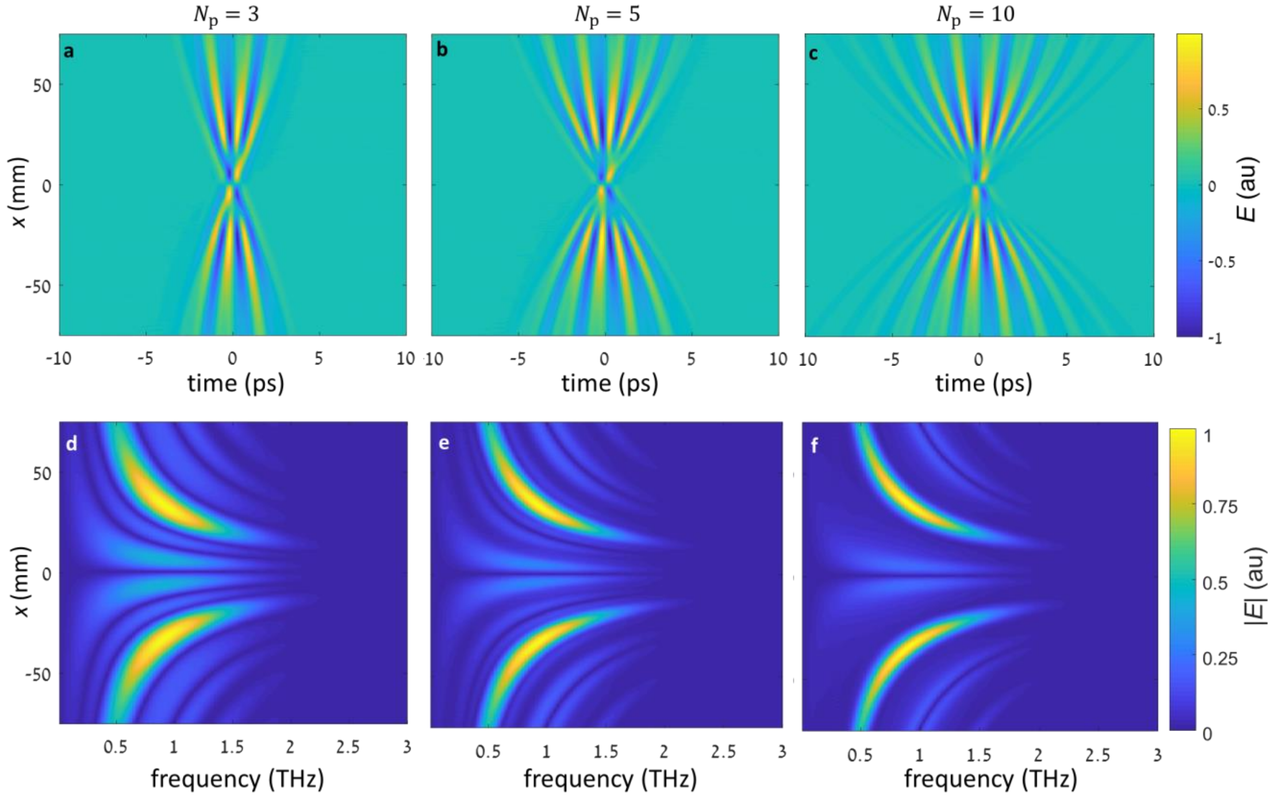

Supplementary Figure 4 Terahertz emission from nonlinear metamaterial photonic crystal with varying number of periods. **a-c** Simulated spatiotemporal emission profile of NLMPCs with constant period $\Lambda = 1$ mm) and varying length $L = 3$mm, 5mm and 10 mm respectively. As a result each plot represents a different N_p. **d-f** Spatiospectral emission profile corresponding to **a-c**.

Supplementary Figure 5 shows an additional set of simulations, in which the length of the metasurface and the NLMPC period (L and Λ) are changed but with a constant ratio, $N_p = 5$. It can be seen, along with Supplementary Figures 4b,e that the number of cycles remains constant for all cases whereas the frequency depended emission angle is changed according to Supplementary Equation 11 and the sinc-like behavior of the spectral pattern is reduced as L increases.

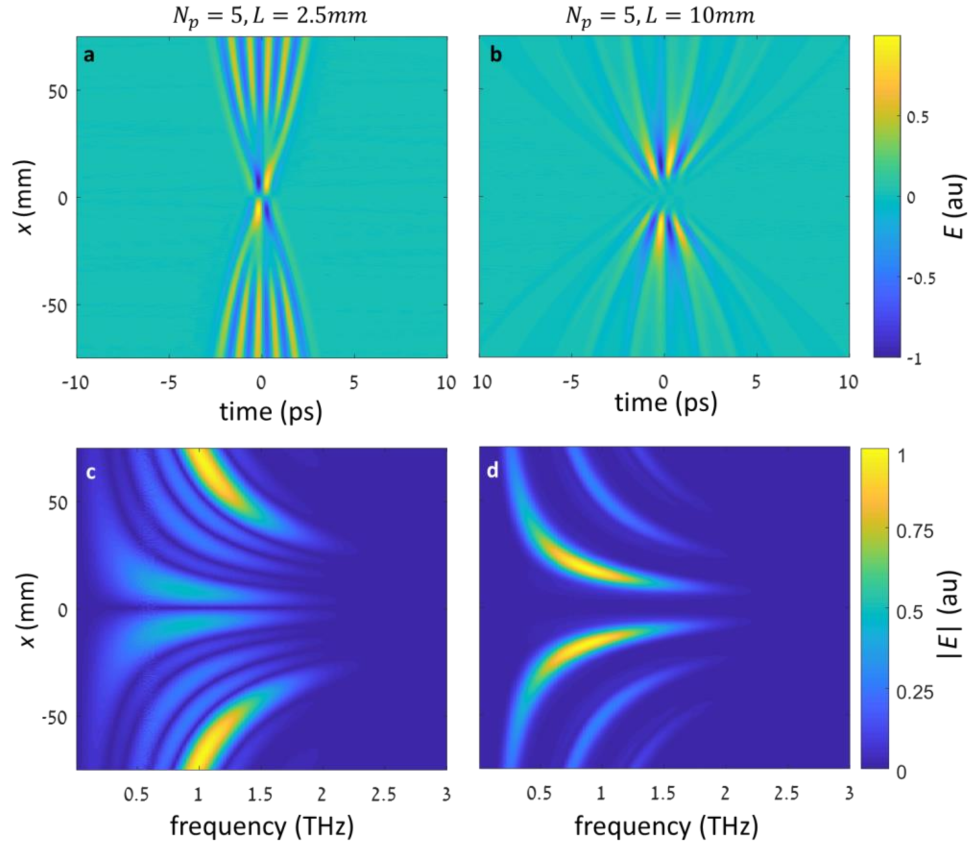

Supplementary Figure 5 Terahertz emission from nonlinear metamaterial photonic crystal with varying period length. **a,b** Simulated spatiotemporal emission profile for constant N=5 with $L = 2.5$ mm and 10 mm respectively. **c,d** Corresponding spatiospectral emission profiles.

Supplementary Note 5

Carrier-envelope shaping of the few-cycle pulse

The use of nonlinear metasurfaces as THz generation platform allows to control the emitted wave by manipulation of the local nonlinear response tensor across the metasurface. In this work we demonstrated it mainly by binary modification of the phase, however, it is possible to control additional nonlinear emission properties. Specifically, the amplitude of the local THz emission

can be controlled, either by local geometrical modification of the single building block, or, more simply, by adjustment of the single-emitters concentration. The local distance between the single emitters can be easily tuned, as long as nearfield effects and collective effects are avoided.

The constant amplitude of the nonlinear response tensor along the NLMPC, is followed by constant carrier envelope amplitude along the few-cycle pulse. Similarly, a modulated amplitude of the NLMPC is translated to carrier-envelope modulation.

For example, Supplementary Figure 6a shows the nonlinear response tensor of amplitude-modified NLMPC with a triangular structure. Supplementary Figure 6b presents the simulated spatiotemporal field distribution of the modified NLMPC. The THz signal emitted at a specific angle, as shown in Supplementary Figure 6c, shows the triangular-modulated few-cycle pulse. A more complex demonstration of this concept is present in Supplementary Figure 6d-f, in which a NLMPC constructed of two modulated periodic structures results with a signal of two consecutive pulses with the predesigned carrier-envelope. This concept opens the door for complex THz pulse shaping and its applications, such as pump-probe experiments, quantum and coherent control, information encoding and more.

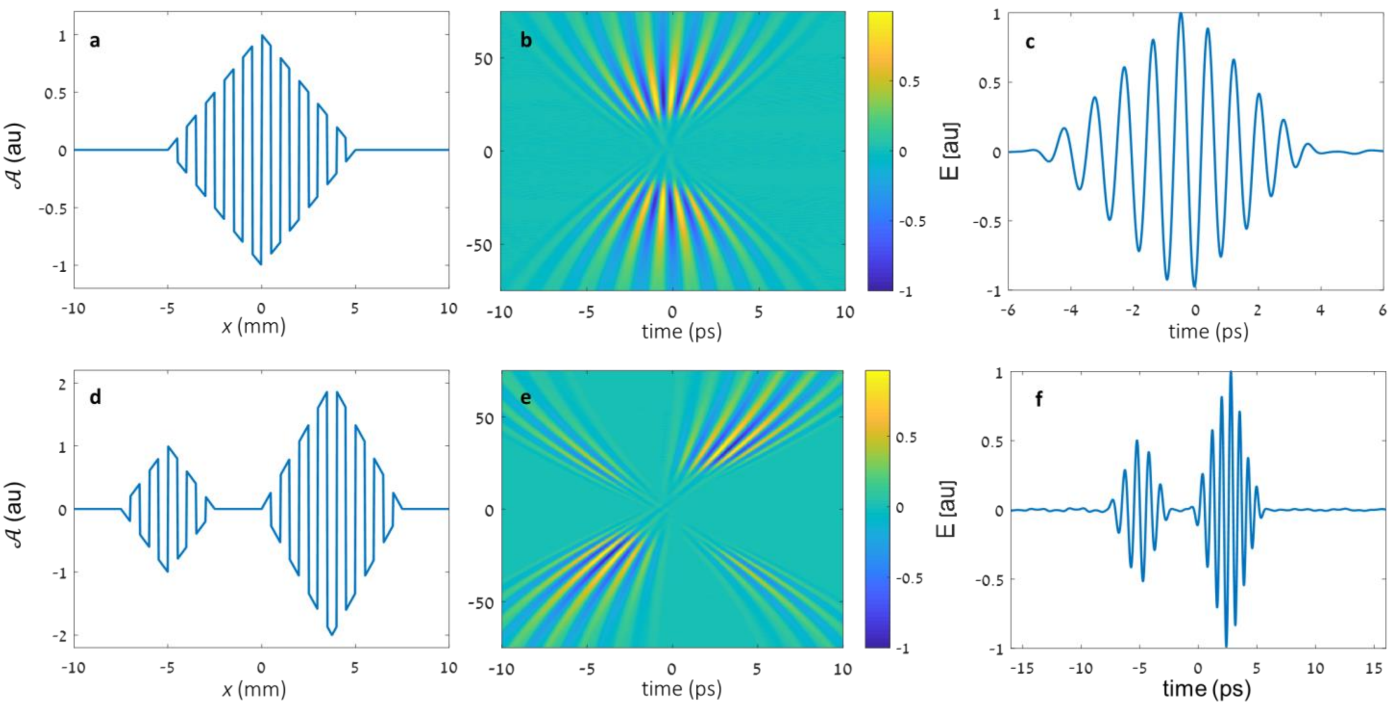

Supplementary Figure 6 Carrier-envelope shaping. **a** Spatial structure of nonlinear response tensor for triangular-modulated NLMPC. **b** Spatiotemporal emission profile of **a**. **c** Signal emitted at 15° ($x = 26.7\ mm$ of the collimated beam), showing a triangular carrier-envelope. **d** NLMPC structure for two consecutive pulses. **e** Spatiotemproal emission profile of **d**. **f** Signal emitted at 15°, showing the corresponding pulse shape.

References:

1. Ziolkowski, R. W. Localized transmission of electromagnetic energy. *Phys. Rev. A* **39,** 2005–2033 (1989).
2. Hellwarth, R. W. & Nouchi, P. Focused one-cycle electromagnetic pulses. *Phys. Rev. E* **54,** 889–895 (1996).
3. Lekner, J. Localized electromagnetic pulses with azimuthal dependence. *J. Opt. A Pure Appl. Opt.* **6,** 711–716 (2004).
4. Lekner, J. Helical light pulses. *J. Opt. A Pure Appl. Opt.* **6,** L29–L32 (2004).
5. Raybould, T., Fedotov, V., Papasimakis, N., Youngs, I. & Zheludev, N. Focused electromagnetic doughnut pulses and their interaction with interfaces and nanostructures. *Opt. Express* **24,** 3150 (2016).
6. Papasimakis, N. *et al.* Pulse generation scheme for flying electromagnetic doughnuts. *Phys. Rev. B* **97,** 201409 (2018).
